# *In-silico* analysis of cis-acting regulatory elements of pathogenesis-related proteins of *Arabidopsis thaliana* and *Oryza sativa*

**DOI:** 10.1371/journal.pone.0184523

**Published:** 2017-09-14

**Authors:** Amritpreet Kaur, Pratap Kumar Pati, Aparna Maitra Pati, Avinash Kaur Nagpal

**Affiliations:** 1 Department of Botanical and Environmental sciences, Guru Nanak Dev University, Amritsar, Punjab, India; 2 Department of Biotechnology, Guru Nanak Dev University, Amritsar, Punjab, India; 3 Planning Project Monitoring and Evaluation Cell, CSIR-Institute of Himalayan Bioresource Technology, Palampur, Himachal Pradesh, India; Birla Institute of Technology and Science, INDIA

## Abstract

Pathogenesis related (PR) proteins are low molecular weight family of proteins induced in plants under various biotic and abiotic stresses. They play an important role in plant-defense mechanism. PRs have wide range of functions, acting as hydrolases, peroxidases, chitinases, anti-fungal, protease inhibitors etc. In the present study, an attempt has been made to analyze promoter regions of PR1, PR2, PR5, PR9, PR10 and PR12 of *Arabidopsis thaliana* and *Oryza sativa*. Analysis of cis-element distribution revealed the functional multiplicity of PRs and provides insight into the gene regulation. CpG islands are observed only in rice PRs, which indicates that monocot genome contains more GC rich motifs than dicots. Tandem repeats were also observed in 5’ UTR of PR genes. Thus, the present study provides an understanding of regulation of PR genes and their versatile roles in plants.

## Introduction

Plants are persistently under the threat of several pathogens like bacteria, viruses, fungi, nematodes and other threats. However plant pathogen interactions are extremely intricate and cause majority of plants to become impervious to the vast majority of pathogens. Further these interactions exhibit specific responses that permit only a few pathogens to colonize and spread disease [1–2]. Early recognition of a pathogen is an indispensable step for disease resistance in plants, which is followed by an activation of a series of defense responses during the interaction. During incompatible interactions, avirulence pathogen proteins (Avr) interact with host resistance (R) genes bringing about a series of defense responses such as: accumulation of Reactive Oxygen Species (ROS); enhancement in abscisic acid (ABA), salicylic acid (SA), jasmonic acid (JA), auxins and gibberellins; synthesis of pathogenesis related (PR) proteins; phytolexins accumulation; and hypersensitive response (HR) induction. Consequently, plants don’t develop disease symptoms and are safe. In case of compatible interactions, the virulent pathogen invades host machinery and results in systemic infection which prompts the development of disease symptoms [1–3].

PR proteins are a collective set of low molecular weight proteins which accumulate under various biotic and abiotic stresses and under specific physiological conditions like pollen development, leaf senescence, fruit development and ripening [4–7]. Such proteins have been considered to perform a number of functions, acting as transcription factors, protease inhibitors, enzymes involved in hydrolysis and many are associated with various metabolic pathways [4, 8]. PRs were isolated for the first time from tobacco leaves (*Nicotiana tabacum*) infected with tobacco mosaic virus [9] and subsequently reported from many other plant species including *A*. *thaliana*, alfalfa, barley, bean, carrot, chickpea, grape vine, maize, pepper, pearl millet, rice, rubber, soybean, sunflower, sorghum, tomato and wheat [10]. PR proteins have been characterized and classified into 17 families based on the sharing of amino acid sequences, serological relationships, and enzymatic or biological activity [11].

PR1 was the first PR protein to be discovered and has a molecular weight of 14 to 17 kD and acts as a molecular marker for systemic acquired resistance response. It has antifungal activity. PR2 proteins are β-1, 3-glucanases, and their molecular mass ranges from 33 to 44 kDa. They comprise large and highly complex gene families involved in pathogen defense as well as a wide range of normal developmental processes. They are induced in response to wounding or infection by viruses, bacteria and fungi. β-1,3-glucanases degrade pathogen’s cell walls by cleaving β-1,3-glucosidic bonds in β-1,3-glucan, a major component of fungal cell wall. PR3 proteins (chitinases) have molecular mass in the range of 15–43 kDa. They cleave the chitin polymers in fungal cell wall, resulting in a weakened cell wall and making fungal cells osmotically sensitive. PR4 proteins are chitin binding proteins having molecular mass between 9–30 kDa. These proteins bind to chitin, and play an important role in enhancing the chitinase activity [6, 12]. Thaumatin-like proteins (PR5) possess molecular mass between 18–25 kDa. These can act as antifungal; glucanase and xylanase inhibitors; and α-amylase and trypsin inhibitors. They are also known to be induced during wounding and by insect feeding; especially by phloem feeding insects [13]. Proteinase inhibitors (PR6) and endoproteinases (PR7) are highly stable defensive proteins of plant tissues that are both developmentally regulated and induced in response to insect and pathogen attacks. PR9 (peroxidase) catalyzes cross-linking of macromolecules in plant cell wall. It also produces free radical like H_2_O_2_ against a wide range of pathogens [6]. PR10 are ribosome inactivating proteins, known to inhibit translation in fungi. These proteins protect plant proteins and other cellular structures during dormancy, salinity or cold stress [14]. PR12 (defensin) are small cysteine rich peptides providing protection against a broad range of organisms. They are known to inhibit protein synthesis, enzyme activity and ion channel function [15]. PR15 and PR16 catalyze oxidation of oxalates by molecular oxygen, yield CO_2_ and H_2_O_2_. They have role in plant development, defense, signaling, differentiation and apoptosis [16].

For a number of PR proteins, activities are known or can be deduced. The majority of PRs (e.g. PR1, PR2, PR3, PR4, PR5, PR7, PR12, PR13 and PR14) possess antifungal activity, whereas, PR8 and PR11 are classified as endochitinases. PR15 and PR16 are oxalate oxidase and oxalate oxidase- like proteins, respectively [4, 16]. However, very little is known about molecular mechanism of gene expression of PR genes. In one study, Lodhi et al., 2008 [17] deduced a relationship among architecture of promoter sequence, positioning of nucleosome and expression of PR-1a in tobacco. Therefore, study of gene expression regulation of PR proteins is a crucial step in understanding the molecular mechanisms of plant defense response. Transcription regulation involves association between transcription factors and particular cis-acting regulatory elements (CAREs) of a specific gene involved in plant defense response [18]. CAREs are short regulatory motifs (5–20 bp) present in the promoter regions of target genes (typically, non-coding DNA). Promoters play an important role in controlling gene expression. Multiple CAREs such as TATA box, GC box, CAAT box contain coupling sites for transcription factors, enhancers and repressor elements required for proper spatiotemporal expression of genes [19]. Cis-acting regulatory elements are essential transcriptional gene regulatory units as they control various stress responses. Recent advancements in such experimental techniques as RNA interference, microarrays, RNAseq and others have allowed identification and investigation of promoter regions of target genes but these techniques are expensive and technically challenging. Therefore, computational methods are being used to search the promoter regions for different cis-elements responsible for the regulation of the genes [18]. Different computer programs can also be used to look for known cis-elements and to study their organization. Such web-based tools as PLACE [20], PlantCARE [21], AGRIS [22], TRANSFAC [23] and PlantPAN [24] have been developed for the analysis of cis regulatory elements in plant genes.

Examination of CAREs within the promoter sequences of PR genes as well as their combinatorial effects, will lead to better comprehension of regulation of their gene expression. Understanding of cis-elements can also allow us to effectively change the expression pattern of a gene in desired way, which further can provide new ways for the plant genetic engineering technology for protection of crops against biotic and abiotic stresses. To the best of our knowledge, no work has been reported on cis-elements of *Arabidopsis thaliana* and *Oryza sativa* PR. Therefore, the present study was planned, to characterize cis-acting regulatory elements (CAREs) of PR classes 1, 2, 5, 9, 10 and 12 with respect to their occurrence and putative role in model plants, *Arabidopsis thaliana* and *Oryza sativa*. We also tried to validate our *in-silico* work with wet lab studies wherever available.

## Materials and methods

### Search for PR genes of *A*. *thaliana* and *O*. *sativa* and their structure analysis

Gene sequences of PR-1, PR-2, PR-5, PR-9, PR-10 and PR-12 of *A*. *thaliana* and *O*. *sativa* were retrieved from The Arabidopsis Information Resource (TAIR) (https://www.arabidopsis.org/) and Rice Genome Annotation Project (RGAP) (http://rice.plantbiology.msu.edu/), respectively. All the gene sequences were verified against Phytozome v 11.0 (https://phytozome.jgi.doe.gov/pz/portal.html) and Plant Genome and System Biology (PGSB) (http://pgsb.helmholtz-muenchen.de/plant/plantsdb.jsp)databases. Comparative analysis of PR gene sequences was performed using MatGAT 2.02 (http://ww3.bergen.edu/faculty/jsmalley/matgat.html) [25] to find percentage similarity and NCBI-Conserved domain database (https://www.ncbi.nlm.nih.gov/Structure/cdd/wrpsb.cgi) [26] to find conserved domains. Chromosome maps of *A*. *thaliana* and *O*. *sativa* PR genes were constructed by Chromosome Map Tools available at TAIR (https://www.arabidopsis.org/jsp/ChromosomeMap/tool.jsp) and Oryza base (http://viewer.shigen.info/oryzavw/maptool/MapTool.do), respectively. The intron/exon organization of splice variants of PR genes of both *A*. *thaliana* and *O*. *sativa* was retrieved from TAIR and Rice Genome Annotation Project respectively. Pearson correlation analysis was performed to find out the relationship between gene lengths of different PRs of *A*. *thaliana* and *O*. *sativa*. Gene Structure Display Server (http://gsds.cbi.pku.edu.cn/) [27] was used to visualize intron/exon organization of genes.

### Retrieval of promoter regions and analysis of cis-regulatory elements

Promoter sequences (1.5 kbp upstream of translation start site) of each *A*. *thaliana* and *O*. *sativa* PR gene under consideration were retrieved from the respective databases i.e. TAIR and RGAP. The tools PlantCare (http://bioinformatics.psb.ugent.be/webtools/plantcare/html/) [21] and AGRIS (http://arabidopsis.med.ohio-state.edu/AtcisDB/) [22] were used for scanning of cis-elements present in promoter regions of PR genes of *A*. *thaliana* whereas, PlantCare and PLACE (http://www.dna.affrc.go.jp/htdocs/PLACE/) [20] were used for identification of cis-elements in promoters of *O*. *sativa* PR sequences. The cis-elements so obtained were compared with each other and discussed in light of literature available. PlantPAN (http://plantpan2.itps.ncku.edu.tw/) [24] was used for the analysis of CpG/CpNpG islands and tandem repeats.

### *In silico* analysis of PR genes expression

Genevestigator (https://genevestigator.com/gv/) [28] was used for performing gene expression analysis.

## Results and discussion

### Search for PR genes of *A*. *thaliana* and *O*. *sativa* and their structural analysis

**Fig 1:**
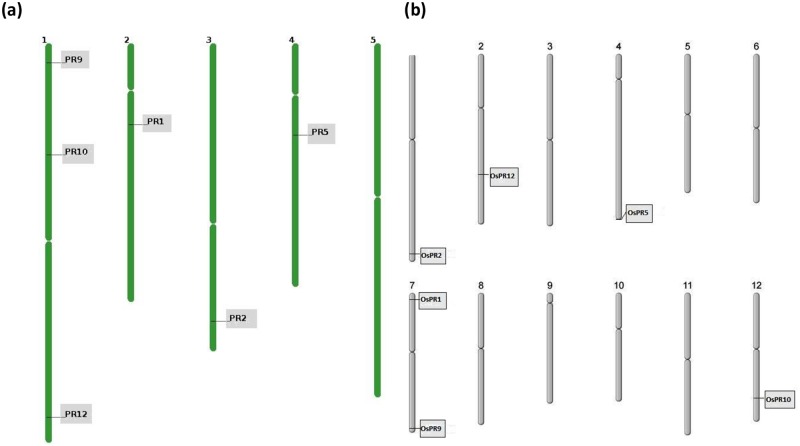
Chromosomal distribution of PR genes in (a) *A*. *thaliana* and (b) *O*. *sativa*.

Characteristics of 6 PR genes of *A*. *thaliana* and *O*. *sativa* retrieved from TAIR and RGAP databases are given in Tables 1 and 2. The gene size of 6 PR genes of *A*. *thaliana* varied from 535 bp in AtPR12 to around 1545 bp in AtPR9 and in *O*. *sativa*, gene size varied from 738 bp in OsPR12 to 2347 bp in OsPR2. Characteristics of each PR gene sequence are given in Tables 1 and 2 and include locus, chromosome number, strand, transcript count, transcript id, gene length, CDS length, number of exons, and protein length. MatGAT tool was used to compare PR gene sequences of *A*. *thaliana* and *O*. *sativa*, which revealed percentage similarity (evolutionary distance) among different PRs to range from 39.4% for PR10 to 67.3% for PR2 (S1 Table). PR10 proteins are coded by multigene families and shows higher inter specific variation. However, all of them possess conserved glycine rich loop in their sequence [29]. PR gene sequences from *A*. *thaliana* and *O*. *sativa* were subjected to comparative analysis for their domain architecture using CDD from NCBI. The analysis revealed the presence of SCP_PR1_like domain in PR1; glycol_hydro_1 domain in PR2; GH64-TLP-SF in PR5; plant_peroxidase_like domain in PR9; SRPBCC in PR10 and gamma-thionin in PR12 of *A*. *thaliana* and *O*. *sativa*, respectively. Outcome of this analysis is the presence of conserved domains in their sequences, which indicate that they are homologs. *In-silico* chromosome mapping of PR genes of *A*. *thaliana* and *O*. *sativa* is presented in Fig 1 (a and b respectively). PR genes of *A*. *thaliana* have been shown to be distributed on 4 out of 5 chromosomes and of *O*. *sativa* are distributed on 5 out of 12 chromosomes. In *A*. *thaliana*, chromosome 1 harbored 3 PR genes, whereas chromosome 5 showed no PR gene. In case of *O*. *sativa*, chromosome number 7 possessed 2 PR genes, while chromosome numbers 3, 5, 6, 8, 9, 10, 11 showed no PR genes.

**Table 1:** Characteristics of PR genes in *A*. *thaliana*.

Gene name	TAIR locus	Chromosome number	Strand	Transcript count	Transcript id	Genomic seq length (bp)	Exons count	CDS length	Protein length (aa)
AtPR1	AT2G14610	2	-	1	AT2G14610.1	760	1	486	161
AtPR2	AT3G57260	3	-	1	AT3G57260.1	1343	2	1020	339
AtPR5	AT4G11650	4	-	1	AT4G11650.1	1291	2	735	244
AtPR9	AT1G05240	1	+	1	AT1G05240.1	1545	4	978	325
AtPR10	AT1G24020	1	-	2	AT1G24020.1	1070	2	468	155
					AT1G24020.2	1666	2	468	155
AtPR12	AT1G75830	1	+	1	AT1G75830.1	535	2	243	80

**Table 2:** Characteristics of PR genes in *O*. *sativa*.

Gene name	*O*. *sativa* XPro ID	MSU loci ID	Chromosome number	Strand	Transcript count	Transcript id	Genomic seq length (bp)	Exons count	CDS length	Protein length (aa)
OsPR1	Os07g0129200	LOC_Os07g03710	7	+	1	LOC_Os07g03710.1	934	1	507	168
OsPR2	Os01g0944700	LOC_Os01g71670	1	-	1	LOC_Os01g71670.1	2347	2	1005	334
OsPR5	Os04g0689900	LOC_Os04g59370	4	+	1	LOC_Os04g59370.1	1376	2	837	278
OsPR9	Os07g0677200	LOC_Os07g48020	7	+	1	LOC_Os07g48020.1	1680	4	954	317
OsPR10	Os12g0555000	LOC_Os12g36830	12	+	1	LOC_Os12g36830.1	1195	2	483	160
OsPR12	Os02g0629800	LOC_Os02g41904	2	+	1	LOC_Os02g41904.1	738	2	243	80

**Fig 2:**
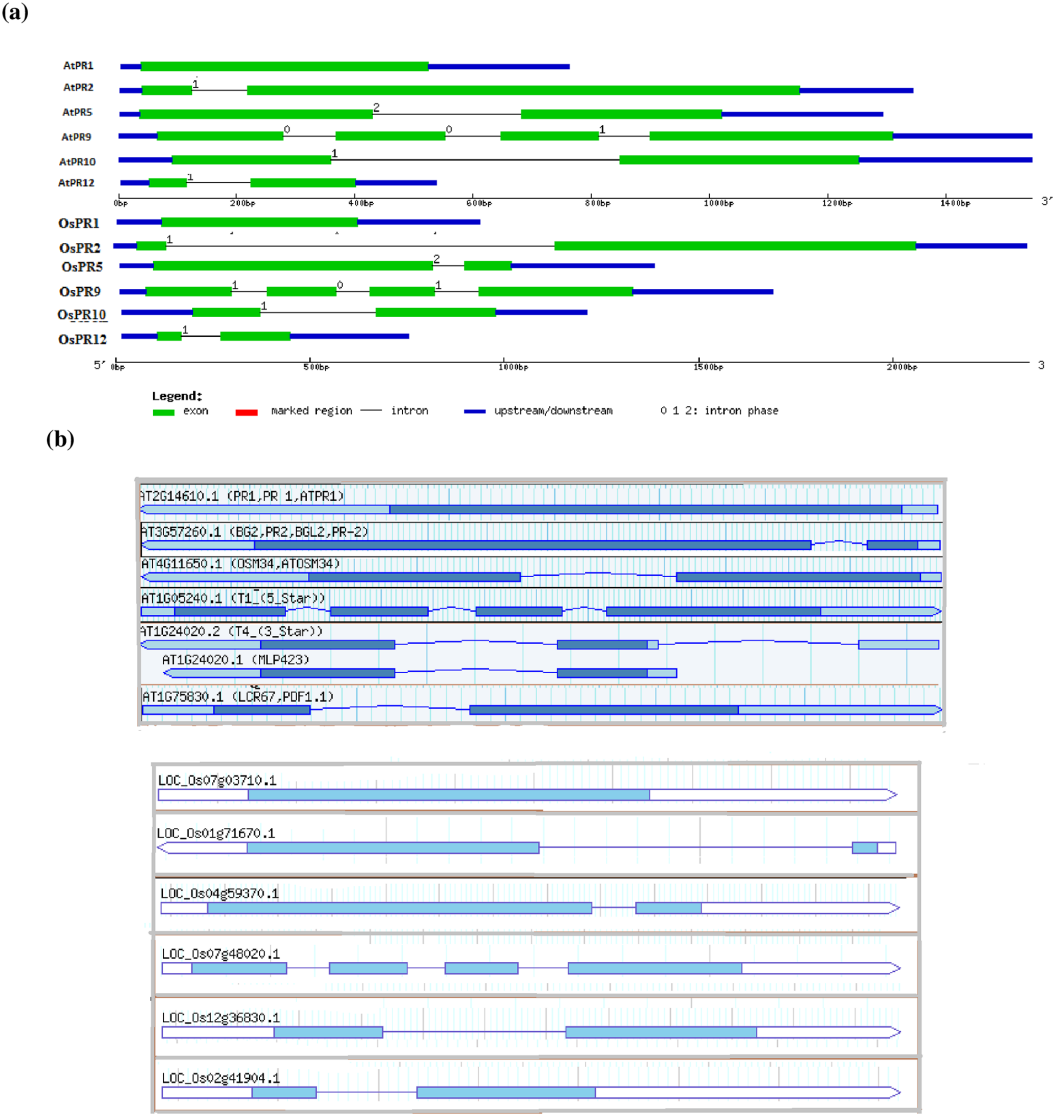
Exon-intron arrangement of PR genes. (a) Blue boxes represent untranslated region. Exons are represented by green boxes and connecting lines represent introns. (b) exon-intron arrangement among splice forms in *A*. *thaliana* and *O*. *sativa*.

The position of exons/introns and intronic phase distribution are important characteristics for gene structure analysis. Introns have been classified into three phases: phase 0 introns are present between two consecutive codons; phase 1 introns are present between the first and second nucleotide of a codon and phase2 introns are present between the second and third nucleotide of a codon [30]. Fig 2a shows the exon-intron and intron phase arrangement of PR genes of *A*. *thaliana* and *O*. *sativa*. PR genes of *A*. *thaliana* and *O*. *sativa* show almost same intronic phase distribution except PR9 gene. Exon count is same in the PR genes of *A*. *thaliana* and *O*. *sativa*. Table 3 shows the position and length of introns-exons organization in *A*. *thaliana* and *O*. *sativa* PR genes. OsPR2 contains intron of 998 nucleotides, whereas AtPR2 intron length is 94 nucleotides. AtPR1 and OsPR1 showed the absence of introns in their gene sequences. No variation has been observed in the exon lengths of *A*. *thaliana* and *O*. *sativa* PR12 gene i.e. in PR12 of both species exon 1 is 64 bp long, while exon 2 is 179 nucleotides long. The intron length however varies slightly like AtPR12 intron is of 107 bp while OsPR12 intron is 100 bp in length. The 5’ and 3’ UTR regions of AtPR12 and OsPR12 varied considerably which contributed to difference in overall length of PR12 gene in two species. Pearson correlation analysis for gene lengths of 5 PR genes (PR1, PR5, PR9, PR10 and PR12) of *A*. *thaliana* and *O*. *sativa* revealed a highly significant coefficient of correlation (r = 0.996 at p<0.001) (S1 Fig). Since the intron length of PR2 gene varied considerably among the two species studied (*A*. *thaliana* intron length 94 bp and *O*. *sativa* intron length 998 bp long), this gene was excluded from correlation analysis. The splice variants of PRs of *A*. *thaliana* and *O*. *sativa* were also analyzed as shown in Fig 2 (b and c); and except in AtPR10, no other splice variant has been observed in the above analysis.

**Table 3:** Distribution and position of introns and exons in *A*. *thaliana* and *O*. *sativa* PR genes.

		PR1	PR2	PR5	PR9	PR10	PR12
		Location (length)	Location (length)	Location (length)	Location (length)	Location (length)	Location (length)
5’UTR before ATG	**At**	**1–34** (**34**)	**1–37** (**37**)	**1–33** (**33**)	**1–65** (**65**)	**1–62** (**62**)	**1–48** (**48**)
	Os	1–114 (114)	1–59 (59)	1–87 (87)	1–68 (68)	1–182 (182)	1–90 (90)
Exon 1	**At**	**35–520 (486**)	**38–122** (**85**)	**34–428** (**395**)	**66–278** (**213**)	**63–249** (**187**)	**49–112** (**64**)
	Os	115–621 (507)	60–135 (76)	88–803 (716)	69–288 (220)	183–357 (175)	91–154 (64)
Intron 1	**At**		**123–216** (**94**)	**429–680** (**252**)	**278–367** (**90**)	**250–587** (**338**)	**113–219** (**107**)
	Os		136–1133 (998)	804–885 (82)	289–380 (92)	358–654 (297)	155–254 (100)
Exon 2	**At**		**217–1151** (**935**)	**681–1020** (**340**)	**368–553 (186**)	**588–868** (**281**)	**220–398** (**179**)
	Os		1134–2062 (929)	886–1006 (121)	381–557 (177)	655–962 (308)	255–433 (179)
Intron 2	**At**				**554–646** (**93**)		
	Os				558–643 (86)		
Exon 3	**At**				**647–812** (**166**)		
	Os				644–809 (166)		
Intron 3	**At**				**813–898** (**86**)		
	Os				810–923 (114)		
Exon 4	**At**				**899–1311** (**413**)		
	Os				924–1318 (395)		
3’ UTR	**At**	**521–760** (**240**)	**1152–1343** (**192**)	**1021–1291** (**271**)	**1312–1545** (**233**)	**869–1070** (**202**)	**398–535** (**137**)
	Os	622–934 (313)	2063–2347 (285)	1007–1376 (370)	1318–1680 (362)	963–1195 (233)	433–738 (305)
Gene length	**At**	**760**	**1343**	**1291**	**1545**	**1070**	**535**
	Os	934	2347	1376	1680	1195	738

### Retrieval of promoter regions and analysis of cis-regulatory elements

**Fig 3:**
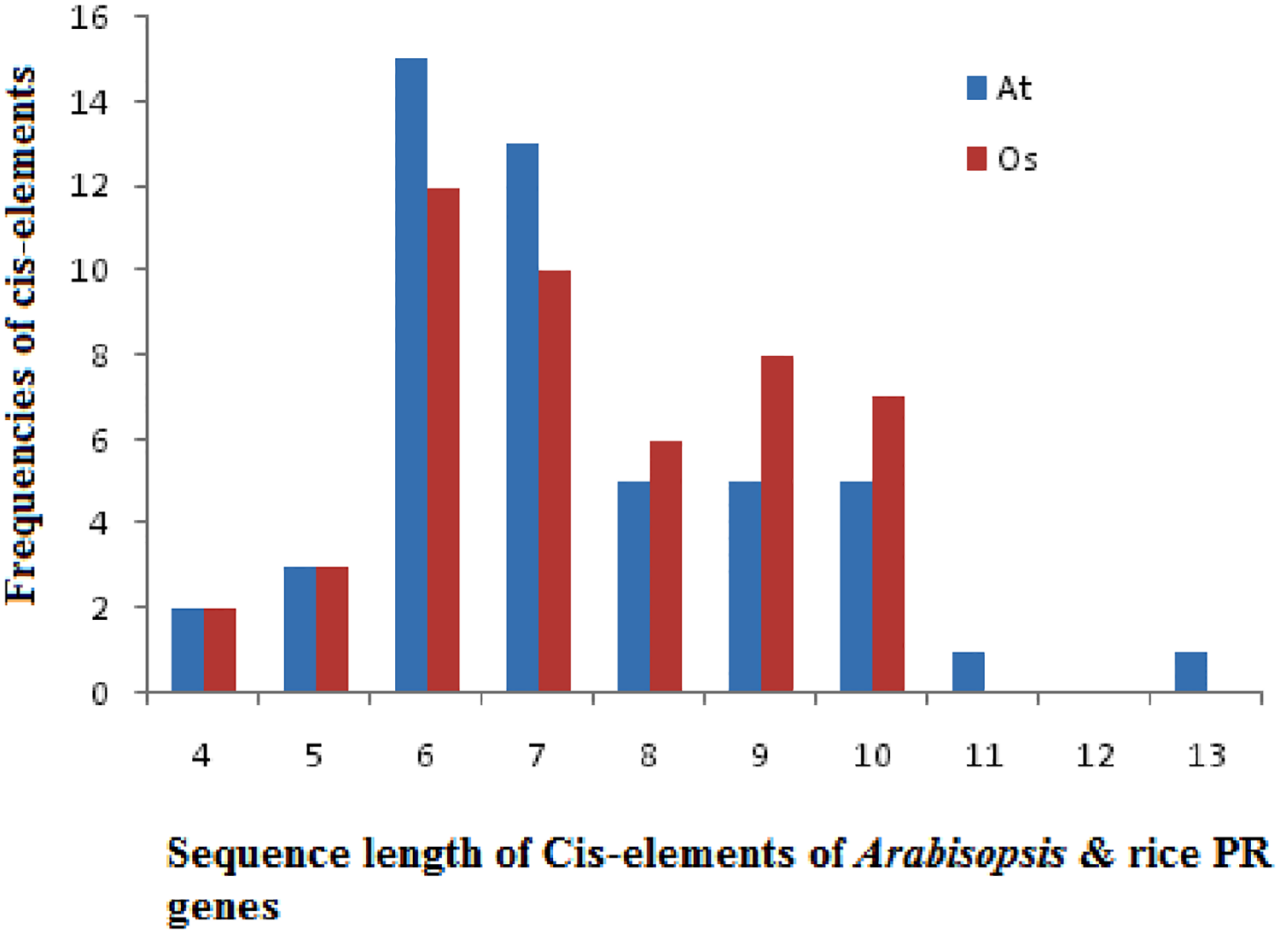
Histogram showing the frequencies of different sequences length of cis-elements in *A*. *thaliana* and *O*. *sativa* PR genes.

**Fig 4:**
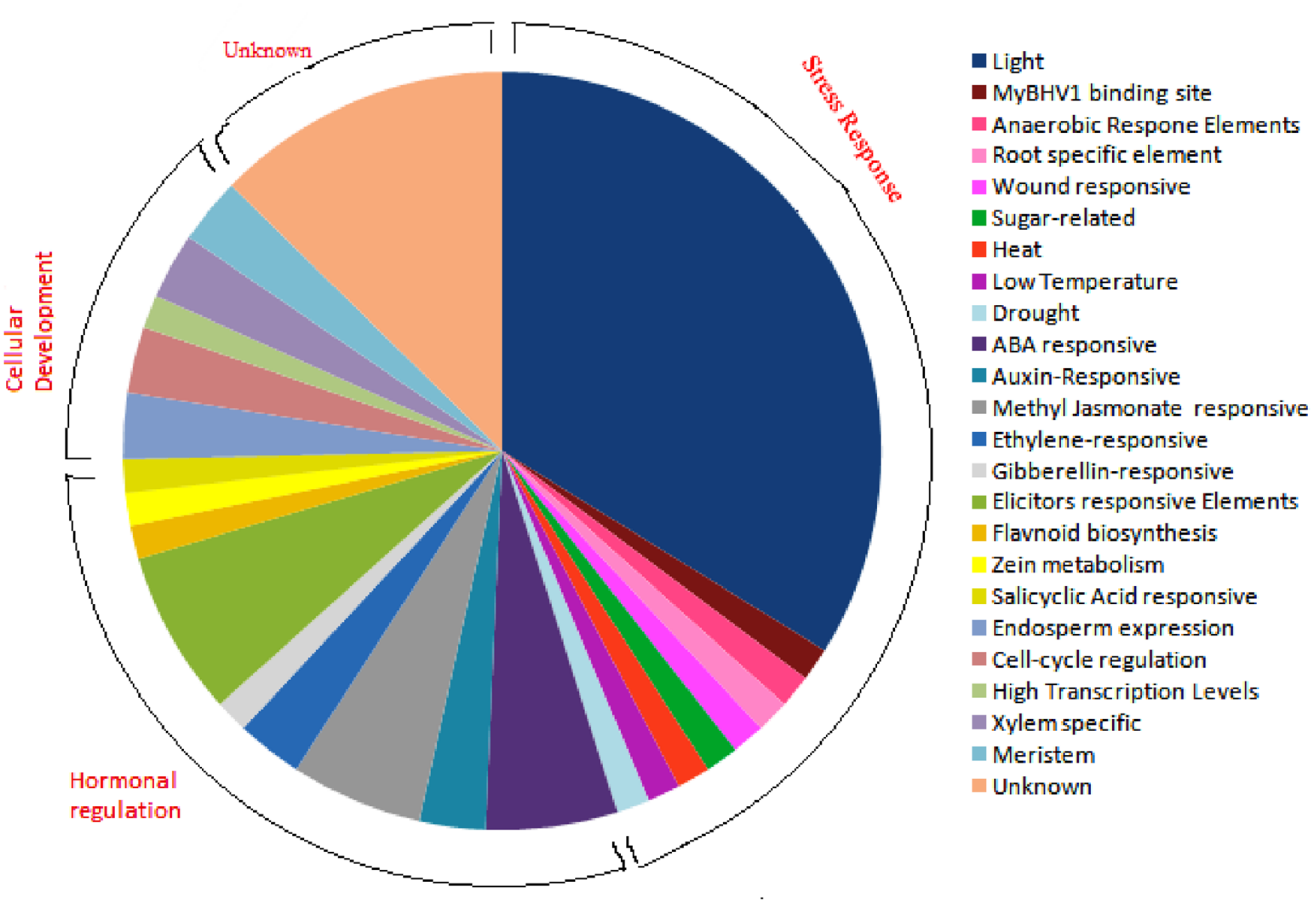
Pie distribution of identified motifs of *A*. *thaliana* and *O*. *sativa* PRs from PlantCARE, based on their biological functions.

**Fig 5:**
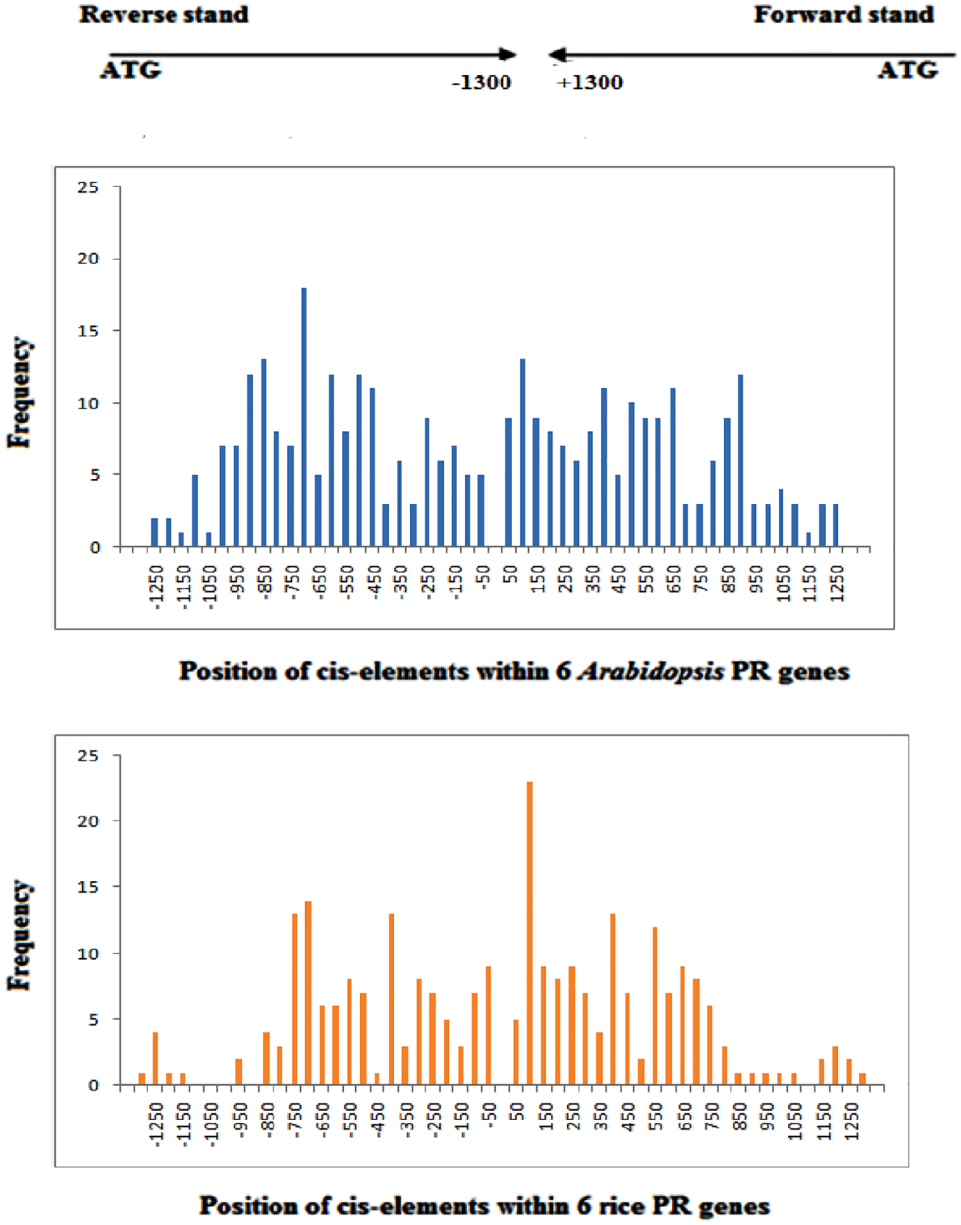
Histogram showing frequencies of occurrence of cis-elements identified using PlantCARE in forward and reverse strands in (a) *A*. *thaliana* (b) *O*. *sativa* PRs.

**Fig 6:**
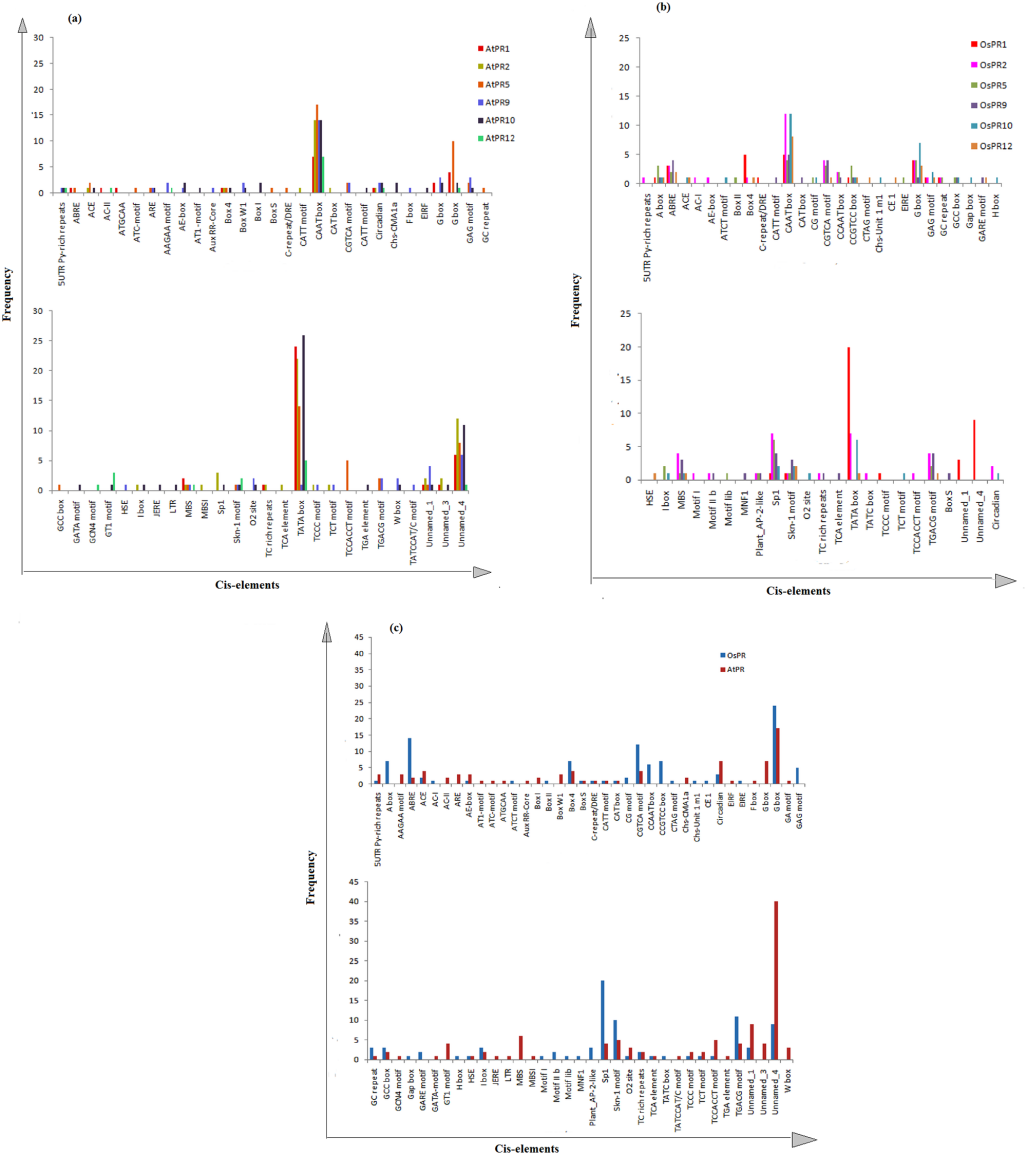
Frequencies of motifs identified using Plantcare in the promoter regions of PR genes of (a) *A*. *thaliana* (b) *O*. *sativa* (c) both *A*. *thaliana* and *O*. *sativa*.

Promoter sequences up to1.5 kbp upstream from the translation start site of each PR gene of *A*. *thaliana* and *O*. *sativa* were scanned using PlantCare program for the identification of cis-acting regulatory elements (CAREs). The study revealed a total of 55 CAREs in *A*. *thaliana* whereas, 48 CAREs were identified in *O*. *sativa*. The length of cis-elements varied from 4–13 bp in *A*. *thaliana* and 4–10 bp in *O*. *sativa* (Fig 3).Majority of cis-elements possessed the length of 6 and 7 bp. Cis-elements are grouped into different functional categories as shown in Fig 4. Stress responsive cis-elements were found to be maximum, and out of those, light responsive elements constituted 34%. Fig 5 depicts the frequency of occurrence of different cis-elements at different positions in the 1.5 kbp of both reverse and forward strands of *A*. *thaliana* and *O*. *sativa* PR genes. In a case of *O*. *sativa*, most of the cis-elements lied between 51-100bp at the forward strand and 351–750 bp at reverse strand. In *A*. *thaliana*, maximum cis-elements lied within the range of 351–650 bp (forward strand) and 701–950 bp (reverse strand). The PR gene sequences of *A*. *thaliana* and *O*. *sativa* were also scanned for the presence of cis-elements by AGRIS and PLACE, respectively. Cis-elements so obtained were compared among themselves, and the results are presented in Table 4. The number of cis-elements including TATA and CAAT box was present in higher amount in *A*. *thaliana* PR genes as compared to that of *O*. *sativa* PR genes. Fig 6 shows the presence of different cis-elements along with their frequencies in *A*. *thaliana* only, *O*. *sativa* only and both *A*. *thaliana* and *O*. *sativa* PR genes. The role of different cis-elements in stress, hormonal regulation and cellular development are discussed in details below.

**Table 4:** Category wise list of cis-elements extracted from 1.5 kbp upstream region of PR genes of *A*. *thaliana* and *O*. *sativa* using PlantCare, PLACE and AGRIS.

Categories based on functions	Sequence	Extracted Cis-element (Tools used)			Presence of cis-element in PR (Tools used)			Specific functions of cis-elements	References
		Plant Care	AGRIS	PLACE	Only *A*. *thaliana* (Plant Care &AGRIS)	Only *O*. *sativa*(Plant Care & PLACE)	Both*A*. *thaliana* &*O*. *sativa*; (AGRIS & PLACE)		
Cellular Functions	CATGCA	_	RY-repeat promoter motif	RYREPEATBNNAPA	-	-	AtPR1, AtPR5, OsPR5	Seed specific regulation	[48]
Stress Response	TTGACC	W-Box/ Box-W1	W-box promoter motif	_	AtPR1,AtPR2, AtPR5,AtPR9, AtPR10, AtPR12	-	-	Fungal elicitor responsive element, wound- responsive	[49]
	GGTTAA	GT1- motif	Box-II promoter site	GT1 consensus	AtPR1,AtPR2, AtPR9, AtPR10, AtPR12	OsPR10	AtPR1, AtPR2, AtPR9, AtPR10, OsPR10	Light responsive	[50]
	AGCCGCC	GCC- box	GCC- box promoter site	GCC CORE	AtPR5	OsPR1, OsPR5, OsPR9,OsPR10	AtPR5, AtPR12, OsPR1	Elicitor responsive element	[51]
	GATAAG	I- box	I- box	-	AtPR2,AtPR5, AtPR9, AtPR10	-	-	Light responsive	[52]
	CACGTG	G-box	G-box promoter motif	-	AtPR1,AtPR5, AtPR9, AtPR10, AtPR12	OsPR1, OsPR2, OsPR5, OsPR9, OsPR10, OsPR12	-	Light responsive	[53]
	CAACGG	CCAAT box	-	MYB2 CONSENSUSAT	-	OsPR2, OsPR5, OsPR9,OsPR10, OsPR12	-	MYBHv1 binding site	[54]
	AGAAAATTCT/AGAAGCTTCT	HSE	HSE binding site motif	-	AtPR9,AtPR10,AtPR12	-	-	Heat responsive	[55]
	AGCCAC	Box S	SORLIP1	-	AtPR5, AtPR12	-	-	Light responsive	[56]
	CCGTCC	A-box/ CCGTCC box	-	PALBOXAPC	-	OsPR12	-	Elicitor or Light responsive	[57]
	TGTATATA	-	SORLREP3	SORLREP3AT	-	-	AtPR1, AtPR5, AtPR10, OsPR5	Light responsive	[56]
	ACTTTG	-	T-box Promoter motif	TBoxATGAPB	-	-	AtPR10, OsPR10	Light responsive	[58]
	TAACTG	MBS	MYB3 binding promoter motif	MYB2AT	AtPR1,AtPR2, AtPR9, AtPR10, AtPR12	OsPR10	AtPR9, OsPR10	MYB binding site involved in drought inducibility	[59]
	CACATG	-	AtMYC2 BS in RD22	E-BOXBNNAPA / MYC-CONSENSUSAT	-	-	AtPR1, AtPR2, AtPR5, AtPR9, AtPR12, OsPR5	Drought -inducible	[60]
Hormonal Regulation	ACGTGGC/ TACGTGC	ABRE	ABRE like binding site motif	-	AtPR1,AtPR5, AtPR9, AtPR12	-	-	ABA- regulated gene expression	[61]
	TGACG	TGACG motif	TGA1 binding site motif	-	AtPR5,AtPR9, AtPR12	-	-	MeJA- responsive element, SA- responsive element	[62]
	TAACTG	MBS	MYB3 binding promoter motif	MYB2AT	AtPR1,AtPR2, AtPR5,AtPR9, AtPR12	OsPR10	AtPR9, OsPR10	ABA- inducible	[59]
	CACATG	-	AtMYC2 BS in RD22	E-BOXBNNAPA / MYC-CONSENSUSAT	-	OsPR5	AtPR1, AtPR2, AtPR5, AtPR9, AtPR10,AtPR12, OsPR5	JA responsive, ABA- inducible,	[60]
	ACACATG	-	DPBF I&2 binding site motif	DPBF CORE DCDC3	-	-	AtPR1, AtPR2, AtPR5, AtPR9, AtPR10, AtPR12, OsPR5, OsPR10	ABA-inducible	[63]

#### Stress responsive cis-elements

Different elements associated with different stress responses such as oxidation, defense, light, cold, drought, dehydration, heat, low temperature, wound were observed in *A*. *thaliana* and *O*. *sativa* PR gene sequences. Oxidative stress is a component of many abiotic and biotic stress conditions such as drought, high temperature stress, UV-B radiation, salinity, metal toxicity, chilling and plant pathogen interactions. PR proteins are strongly induced in response to different types of stresses including wounding or infection by pathogens. Under normal conditions, production of Reactive oxygen species (ROS) in plants is very low and under various environmental stresses, ROS is drastically increased in plants disturbing the normal balance of superoxide anion (O_2_−), hydroxyl radical (·OH), singlet oxygen (1O_2_) and hydrogen peroxide (H_2_O_2_) in the intracellular environment [31, 32]. Microarray expression analysis of *A*. *thaliana* revealed a range of cis-elements responsive to different types of ROS. Such elements have been categorized into two different categories: common ROS-related e.g. TATCCAT/C-motif, GCN4_motif and G-box and ROS-specific element like W-box [33]. Some other elements like H-box, ethylene-responsive GCC elements, salicylic acid, ethylene, abscisic acid and calcium are known to contribute to the response to oxidative damage in *A*. *thaliana* [34].

TATCCAT/C-motif is an amylase element representing sugar repression responsiveness. It plays an important role in GA-regulated expression. AtPR9 (peroxidase) shows the presence of this motif. In *A*. *thaliana*, peroxidases play an important role in generating H_2_O_2_ during the defense response and also provide resistance against a wide range of pathogens. Peroxidases also play a vital role in leaf expansion [35].

GCN4_motif (TGTGTCA) is an essential cis-element required for an endosperm specific gene expression. AtPR12 shows the presence of GCN4_motif. AtPR12 has a role in protecting germinating seeds and developing seeds [36].

G-box (CACGTG) element is involved in response to light, abscisic acid, methyl-jasmonate and anaerobiosis and has a role in ethylene induction as well as in seed specific expression. It is also known as ABRE (ABA-responsive element) [37, 38]. It has been shown to be present in all *A*. *thaliana* PR genes except in AtPR2 and in all *O*. *sativa* PR genes (Fig 5a and 5b).

W box (TTGACC) is an elicitor responsive cis-element, present in AtPR 9 and AtPR 10. W-boxes are found to interact with transcription factors belonging to WRKY family. W box regulates the expression of defense-related (PR10) genes and has role in biotic and abiotic stresses; seed dormancy; senescence etc [39–40]. During stress response, AtPR10 is induced by ABA, ethylene, jasmonic acid and salicylic acid. This gene may be induced by ROS and may act as a protinase against cellulases and pectate lyases of the pathogen [29]. Increase in ROS level especially H_2_O_2_ has been shown to increase the PR10 in plants [41]. The presence of W box in AtPR9 indicates its role in senescence [40].

In addition to the above mentioned responsive elements, other oxidative stress responsive elements like AREs, ethylene-responsive GCC elements, ERE and H-box are also present in promoters of PR gene sequences (Fig 5a and 5b). AREs (Anaerobic responsive elements) are essential for anaerobic induction, present in AtPR5, AtPR9 and AtPR10. AREs are bipartite elements consisting of GC and GT motifs. GT motif resembles AtMYB2 transcription binding site, which is drought and low oxygen induced element [42].GCC-box, ethylene-responsive element is necessary for high-level jasmonate-mediated regulation of PR12 expression during plant defense response [43–44]. GCC element is also associated with the expression of many genes involved in different kinds of abiotic and biotic stresses. H-box is a root specific regulatory element present in AtPR9 and OsPR10. It regulates defense genes by elicitors and other stress stimuli [45]. The TGACG motif, also known as ‘as1 element’, is another well characterized cis-element present in plants. TGACG motif is methyl jasmonate responsive element present among *A*. *thaliana* and *O*. *sativa* PR gene sequences. The transcription of TGACG mediated PR sequences is regulated by binding of BZIP TGA factor to TGACG element [46]. TC- rich repeats are seen in AtPR1, AtPR2, OsPR2 and OsPR9 and have role in stress and defense responsiveness. Whereas, G-box [47] and TATCCAT/C-motif are also involved in regulating defense responses.

A number of cis-elements associated with light stress include ACE, AE-box, ATC-motif, ATCT motif, Box I, Box II, BoxW1, Box4, Box S, CATT motif, CG motif, Chs-CMA1a, Chs-unit 1 ml, G box, GA motif, GAG motif, GATA-motif, Gap box, GT1 motif, I-box, MNF1, Sp1, TCCC motif and TCT motif. Among these some elements such as ACE, AE-box, Box4, Box S, CATT motif, G box, I-box, Sp1, TCCC motif and TCT motif are present in both *A*. *thaliana* and *O*. *sativa* PRs, whereas Chs-unit 1 ml and Gap box (OsPR10); Box II (OsPR5); HSE (OsPR12); and C repeat/ DRE (OsPR1) are present only in *O*. *sativa*. GATA-motif and Chs-CMA1a motif (AtPR10); I-box and Sp1 (AtPR2); Box S (AtPR5); C repeat/ DRE (AtPR5); LTR (AtPR10); and MBS (AtPR1, AtPR2, AtPR5, AtPR9 and AtPR12) are present only in *A*. *thaliana* PRs.

#### Cis-elements in hormonal regulation

Motifs involved in hormonal regulation were found to be second largest in number after stress responsive motifs present in PRs. Few motifs such as ABRE (abscisic acid), CGTCA and TGACG (methyl-jasmonate), GCC-box (ethylene), TCA element (salicylic acid) (Fig 5c) were present in both *A*. *thaliana* and *O*. *sativa* PRs. Some motifs were only limited to *O*. *sativa* PR genes, like TATC and GARE motifs are gibberellin-responsive elements present in OsPR2 and OsPR12, respectively. Abscisic acid responsive elements such as motif II b (OsPR2 and OsPR9), motif lib (OsPR5) and CE1 (OsPR12) were also observed only in *O*. *sativa* PRs. PLACE tool shows the presence of E-box and DPBF CORE DCDC3 motifs in OsPR5. Auxin responsive elements, AuxRR-core and TGA- element were found in AtPR9 and AtPR10, respectively. A MeJA responsive element, JERE motif was found in AtPR10. The AGRIS tool revealed the presence of CACATG motif, which is the binding site for MYC2 transcription element and has a role in jasmonic and abscisic acid signaling have been reported in all *A*. *thaliana* PRs [64].

#### Role of cis-elements in cellular development

Motifs involved in cellular development are relatively less in number as compared to hormonal and stress responsive elements. Fifteen types of motifs related to cellular development were found. These cis-elements include AC-I, AC-II, 5’UTR Py-rich stretch, CAT box, F-box, GCN4_motif, Skn-1-motif, O2-site, circadian, CCGTCC box, motif I, Plant AP-2 Like, H-box, ARE, MBSI (Fig 6a and 6b). AC-I and AC-II are involved in xylem specific expression. AC-I is present in OsPR2, whereas, AC-II is found in AtPR1 and AtPR12. 5’ UTR Py-rich stretch (AtPR1, AtPR12 and OsPR2) and Plant AP-2 Like (OsPR2, OsPR5 and OsPR9) confer high transcription level. CAT box (AtPR2 and OsPR9) and CCGTCC box (OsPR2, OsPR9 and OsPR12) have role in meristem-specific activation, F-box (AtPR9) confers role in cell cycle regulation (Fig 5a and 5b). GCN4_motif (AtPR12) and Skn-1-motif (AtPR5, AtPR9, AtPR10, AtPR12 and in all *O*. *sativa* PRs except OsPR5) are involved in endosperm expression. O2-site (OsPR10, AtPR9 and AtPR10) involved in zein metabolism and circadian motif (present in all AtPRs except AtPR12, OsPR2 and OsPR10) has role in circadian control. Motif I (OsPR2) and H-box (OsPR10) are root specific regulatory motifs. ARE (AtPR5, AtPR9, AtPR10) is essential for anaerobic induction and MBSI (AtPR2) is involved in flavonoid biosynthesis gene regulation (Fig 5c).

#### Calcium responsive cis-elements

**Fig 7:**
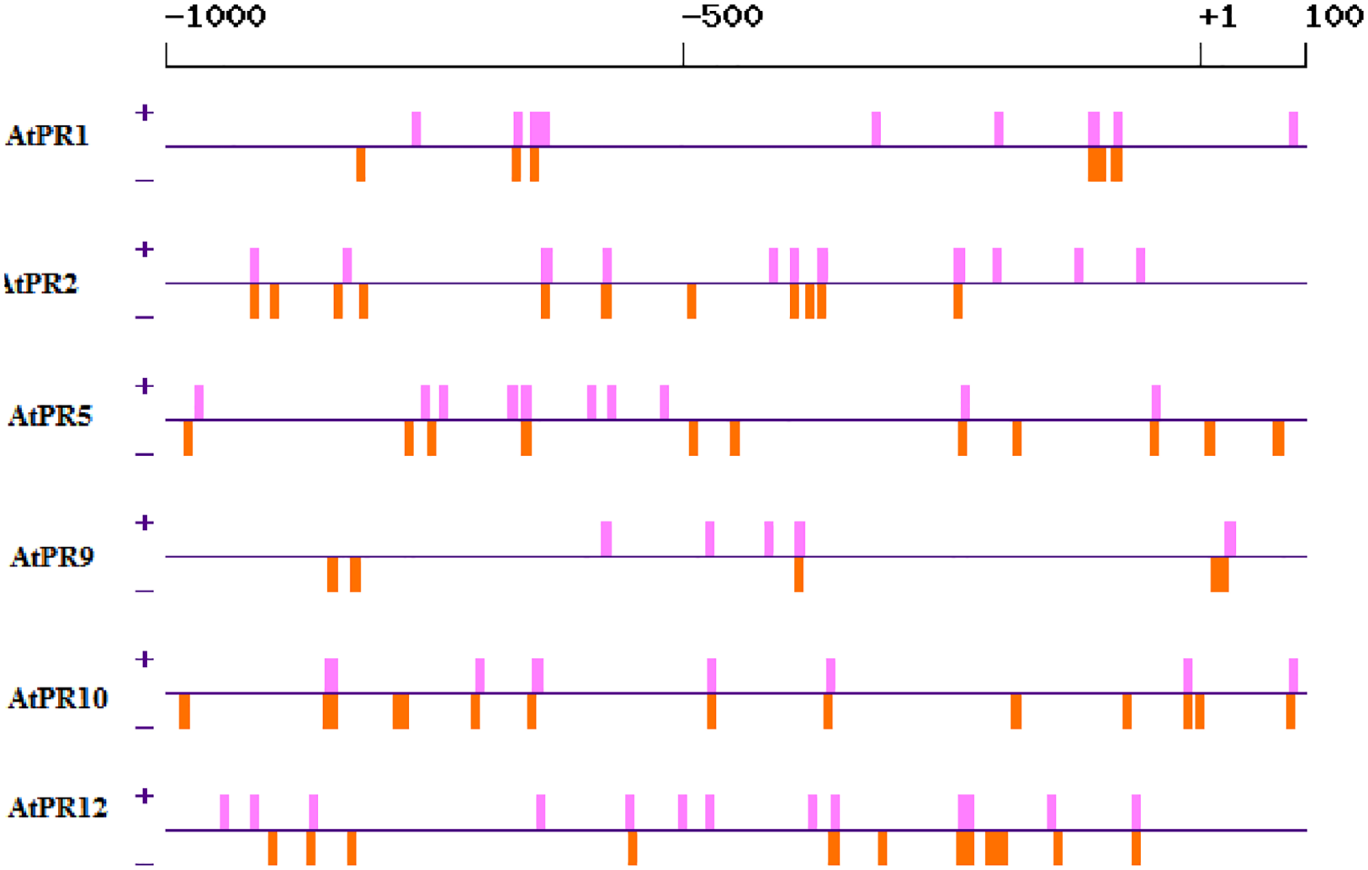
Distribution of identified calcium-responsive cis-element (ABRE) within *A*. *thaliana* PR genes.

Calcium (Ca^2+^) is an intracellular regulator, consequential for many plant biological functions. Ca^2+^signaling is a paramount mechanism evolved in plants to defend themselves against pathogens. It is required for inducing defense-related genes and hypersensitive cell death. Calmodulins (CaM) interact with specific TF (WRKY, MYB, and NAC) families, and regulate the expression of defense genes, but the direct role of CaM in regulating plant defense genes has not been studied so far [65,66]. There are few specific promoter motifs which are regulated by calcium. These include ABRE or ABRERATCAL (Abscisic Acid Responsive—Element), C-Repeat/ DRE (Drought-Responsive Element), Site II, CAM box, CRT and W-box [67, 68] ABRERATCAL (MACGYGB where M = C/A, Y = T/C, B = T/C/G) is the binding site harbored by ABA-induced gene promoter [69]. In the present study, presence of calcium responsive cis-elements was identified by using PlantPAN. We observed the presence of ABRE related elements in all the *A*. *thaliana* PR sequences (Fig 7), whereas, no ABRE like elements was observed in *O*. *sativa* PR sequences.

#### Analysis of conservation of cis-elements in promoter regions of PR genes

Analysis of Phylogenetic conservation of sequences involves the identification of conserved motifs across the genes. The goal of this work was the identification of cis-regulatory sequences conserved in promoters of PR genes of *A*. *thaliana* and *O*. *sativa*. PlantCare data was analyzed to reveal the conserved sequence motifs in promoters of *A*. *thaliana* and *O*. *sativa* PR genes (S2 Table). CAAT and TATA box act as binding sites for transcription factors. CAAT box, important in core promoter activity is almost conserved in all PRs of *A*. *thaliana* and *O*. *sativa*, whereas, TATA box is conserved in most of the PRs except PR5 and PR9. Zuo and Li (2011) also showed the presence of TATA-less promoters in plant genome [70], which indicates that TATA box is not conserved in all the plant genomes. G-box is conserved in all PR promoter regions of *A*. *thaliana* and *O*. *sativa* except PR2. Ishige et al., [71] examined 11 different G-box tetramers in regulation of GUS gene expression and found each G-box sequence influenced gene expression in different ways. One of the G-box sequences, G-box 10 was shown to confer high level constitutive expression in roots, leaves and seeds. MBS cis-element is present in PR2, PR5, PR9 and PR12. MYB transcriptional factor requires MBS for the gene expression of drought inducible genes. CGTCA motif is also found to be conserved in PR5 and PR9 of *A*. *thaliana* and *O*. *sativa*, involved in methyl jasmonate (MeJA) responsiveness. It activates series of defense mechanisms in response to different abiotic stresses like drought, salinity and low temperature. MeJA motif in the 5’ UTR of PR genes infers a possible role in pathogen stress or wound responses. PR1 and PR5 show the presence of ABRE motif, a positive regulator of abscisic acid signaling under drought stress and high salt condition in the vegetative tissues of plants.

There are few CAREs which are unique to *A*.*thaliana* PR proteins (S2 Table). CAREs like A box and CCGTCC box are development related motifs involved in activation of meristem specific expression. GARE and TATC box are cis-acting regulatory elements involved in gibberellin responsiveness. CE1, Motif II b and motif lib are abscisic acid responsive elements unique to *A*. *thaliana* PRs. Cis-elements present only in PRs of *O*. *sativa* are mentioned in (S2 Table). F box is cis-element conserved in PR9; involved in regulating plant defense responses in response to biotic and abiotic stresses. GCN4 motif present in PR12 has role in endosperm specific gene expression.

### Tandem repeats and CpG/CpNpG analysis by PlantPAN

The eukaryotic genome has a wide number of DNA repeats and these repeats have a role in genome evolution [72]. Repetitive DNA may be interspersed in a tandem configuration throughout the genome or may be restricted at some specific location. DNA tandem repeats according to their repeated unit length can be classified into three groups: (i) microsatellite—repeat unit less than 9 nucleotide in length (ii) minisatellite—with 6–100 bp (usually around 15 bp) repeats (iii) megasatellite—tandem repeats of longer units, with length more than 135 nucleotides [18, 73]. Microsatellites are codominant, abundant, multi-allelic, so can be used as molecular markers, in linkage mapping and gene tagging [74].The 1.5kbp upstream promoter region of PR genes revealed the presence of DNA tandem repeats. We found tandem repeat units in three AtPRs (AtPR1, AtPR9 and AtPR12) and one in OsPR (OsPR1) (Table 5). AtPR1 and AtPR9 contain mononucleotide repeats with a repeat size of 1 nucleotide. AtPR12 contain minisatellite. OsPR1 has tetranucleotide repeat with the repeat size of 4 nucleotides. Variation in length of tandem repeats in promoter region could be due to numerical changes like addition and deletion of transcription factor binding sites [75].

**Table 5:** List of PR genes showing tandem repeats within *A*. *thaliana* and *O*. *sativa* promoter regions.

S.No	Gene name	Start	End	Period size	Copy number	%matches	%Indels	score	Entropy (0–2)	Repeat sequence
1	AtPR1	266	298	1	33	93	0	57	0.2	T
2	AtPR9	59	102	1	44	83	0	52	0.58	T
3	AtPR12	1077	1112	18	2	94	0	63	1.38	TTTTCTTCGCTGCTCTTG
4	OsPR1	451	482	4	8	92	0	55	1	TAAT

Epigenetic modifications like DNA methylation, chromatin remodeling and histone modification are heritable changes in gene expression which influence the phenotype [76]. Among these, DNA methylation is important and affects gene expression in plants and animals [77]. DNA methylation occurs at cytosine base, within CpG dinucleotide or may occur at CpNpG (N = A, C or T) sites [78]. CpG rich regions are named as CpG islands and to classify a genome region as CpG island, three conditions must be fulfilled (i) GC content should be above 50% (ii) length of CpG/CpNpG region should be greater than 200 bp (iii) ratio of observed-to-expected CpG dinucleotide number should be above 0.6 [18]. CpG islands are present at or near the gene’s transcription start site and they may regulate the tissue-specific gene expression [79]. DNA of plant species has been shown to contain more CpG dinucleotides than human DNA [80]. Methylation of cytosine at CpG islands has been shown to restrict the access of promoter region of genes to their transcription factors hence, preventing their expression [81]. Cytosine methylation patterns are not static; they change substantially with developmental state or with environmental conditions across the plant genome [82]. DNA methylation has also been shown to play an important role in plant embryogenesis, seed development, in regulating an immune response to infection by pathogens, environmental adaptation and stress resistance. Defects in methylation can cause defect in embryogenesis like abnormal cell division and seed viability reduction, developmental retardation, reduced plant size and partial sterility [77, 83–84]. CpG/CpNpG analysis revealed the occurrence of CpG/CpNpG islands in the second half of the promoter region (towards 3’ end) of all the OsPRs except in OsPR2 but none is identified in AtPRs (Table 6). The absence of CpG/CpNpG islands in the OsPR2 might be due to spontaneous deamination in the germline during evolution [85]. The study performed by Ferguson and Jiang [86] also showed that monocot genome contains more GC-rich content than dicots. The absence of CpG islands in *A*. *thaliana* as compared to *O*. *sativa* genome may be due to the difference in codon usage, GC-biased gene conversions and mutational biases prevalent in two species [87].

**Table 6:** CpG/CpNpG in the promoter region of *O*. *sativa* PRs.

S.No	Gene name	Start	End	Length	Strand	G+C frequency	CpNpG Ratio	AT skew	GC skew
1	OsPR1	649	1500	852	+	0.55	1.3	0.02	0.02
2	OsPR5	735	1500	766	+	0.57	0.93	-0.1	0.06
3	OsPR9	746	1500	755	+	0.5	0.86	0.02	0.06
4	OsPR10	696	1500	805	-	0.49	0.57	0	-0.07
5	OsPR12	65	1500	1436	+	0.53	0.96	-0.08	-0.03

### *In silico* analysis of PR genes expression

**Fig 8:**
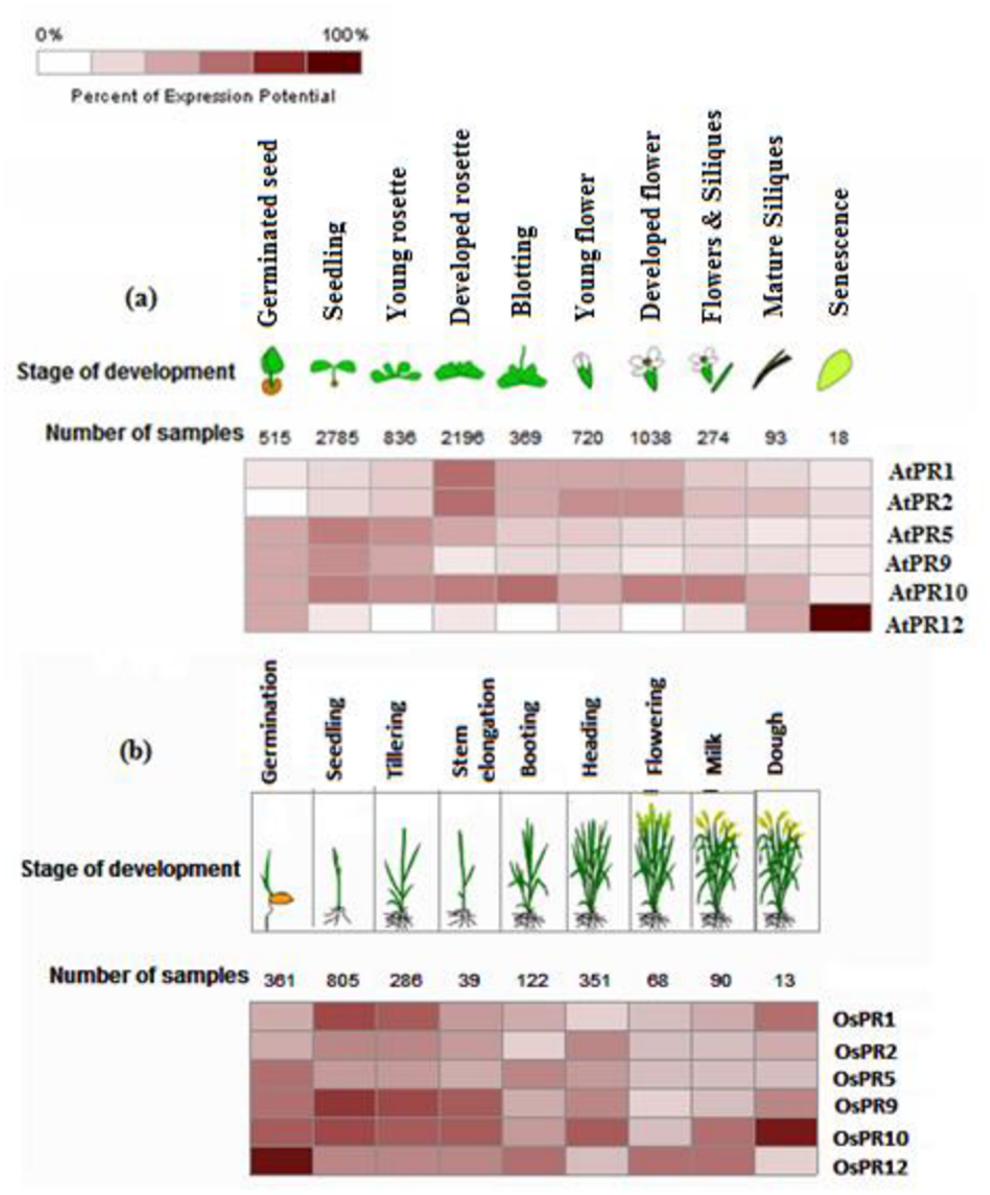
Heat map representation of expression analysis of PR genes at different developmental stages of (a) *A*. *thaliana* (b) *O*. *sativa*.

Meta-analysis of Genevestigator microarray datasets was performed on *A*. *thaliana* and *O*. *sativa* PR genes (Fig 8a and 8b). AtPR10 was a highly expressed gene in almost all stages of development except senescence, where, AtPR12 showed the highest expression during the senescence stage. AtPR1 and AtPR2 expression was maximal during developed rosette stage and minimal during the seedling stage. During the germinating seed stage, AtPR5, AtPR9, AtPR10 and AtPR12 showed same level of expression. Analysis of expression patterns of OsPRs revealed that OsPR10 showed the highest expression at dough stage whereas, OsPR12 appeared to be highly expressed at germination stage. OsPR9 and OsPR10 shared a similar kind of expression at many development stages except at heading, milk and dough stages. OsPR2 and OsPR12 also shared low expression during seedling, tillering and stem elongation stages.

**Fig 9:**
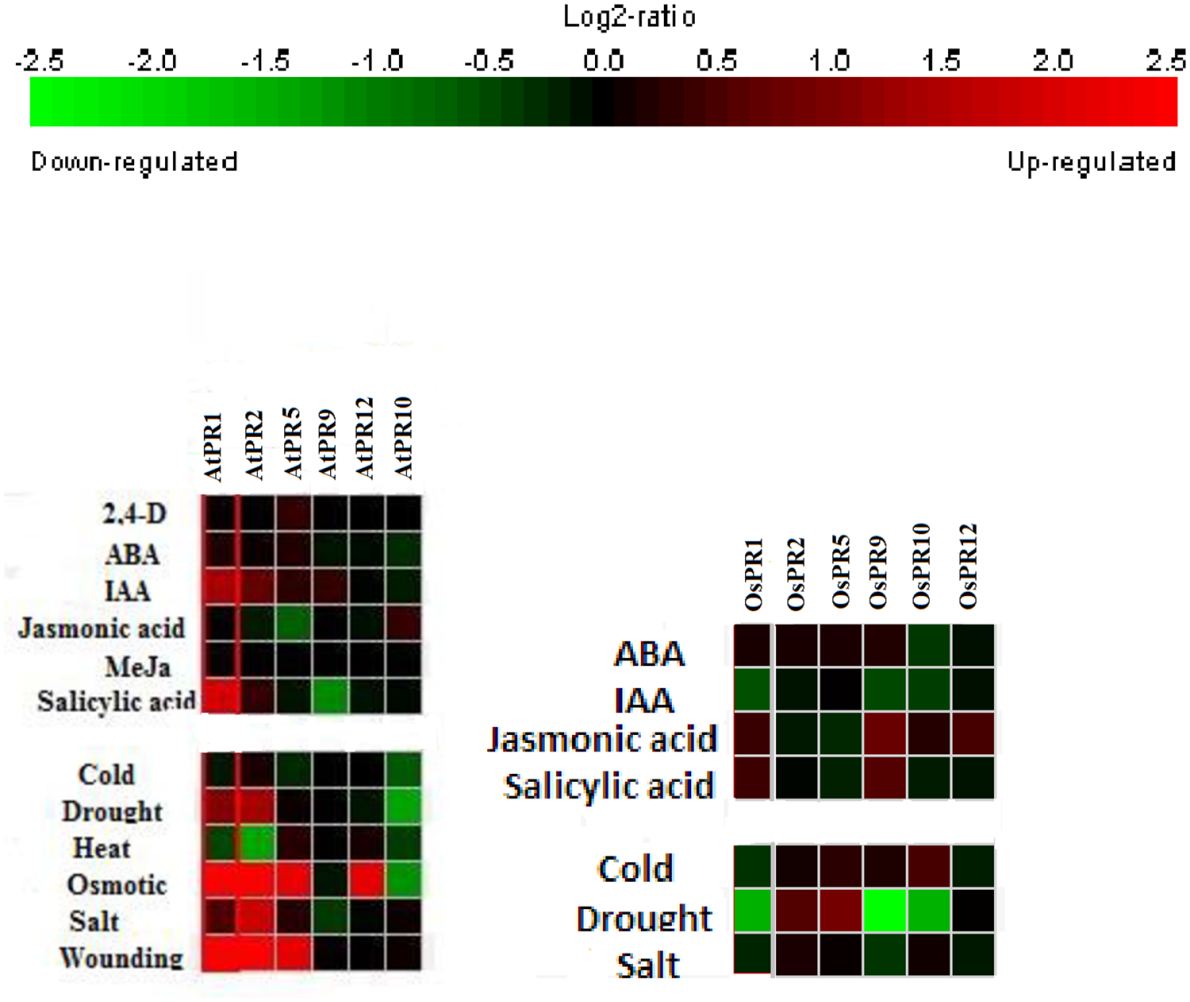
Heat map representation of expression analysis in response to hormonal and abiotic stresses of (a) *A*. *thaliana* (b) *O*. *sativa* PR genes based on microarray data available at Genevestigator.

Expression analysis of *A*. *thaliana* and *O*. *sativa* PR genes in response to hormonal treatments and abiotic stresses were also investigated by Genevestigator (Fig 9a and 9b). Based on microarray data available, it has been observed that in response to hormonal treatments, AtPR1, AtPR2 and AtPR5 were highly up-regulated by external application of salicylic acid, whereas; AtPR9 and AtPR10 were down-regulated. AtPR1 and AtPR2 were also up-regulated by IAA and ABA, respectively. In case of *O*. *sativa* PRs, OsPR9 and OsPR12 were minimally up-regulated by the application of jasmonic acid.

Furthermore, the expression profiles of AtPR genes in response to different abiotic stresses (cold, drought, heat, osmotic, salt and wounding) were also analyzed. Up-regulation was observed in AtPR1 and AtPR2 under drought; AtPR1, AtPR2, AtPR5 under osmotic stress and in wounding; and in AtPR2 under salt stress. Down-regulation was observed in AtPR1 and AtPR2 under heat stress; AtPR9 under salt stress and AtPR10 under cold stress. Expression profiles of OsPRs for cold, drought and salt were also retrieved. OsPR5 was highly and OsPR2 was minimally up-regulated, whereas OsPR1, OsPR9 and OsPR10 were down-regulated under drought stress. Under cold stress, OsPR10 was slightly up-regulated and OsPR1 was minimally down-regulated.

The present study, involved identification of different cis-elements associated with biotic and abiotic stresses present in 5’ UTR sequences of PR genes of *A*. *thaliana* and *O*. *sativa*. The effort has been made to validate the functions of PR’s cis-elements with the microarray data and with literature, wherever it is available. Microarray data indicated stage-specific expression of many AtPRs and OsPRs during different development stages. In case of *O*. *sativa* PRs, OsPR5 is induced under drought stress and in germinating stage; this may be due to the presence of AtMYC2 and RY-element cis motifs, respectively. Microarray data also revealed slight up-regulation of OsPR12 gene during the flowering stage, this can be linked up with the presence of A-box, cis-regulatory element involved in specific day-length to control flowering in *O*. *sativa* [88]. The oxidative stress responsive cis-element like ABRE has been shown to up-regulate OsPR2, OsPR5 and OsPR10 under stresses like drought and cold providing clue towards its diverse roles [89]. Various elicitor responsive elements like GCC box and W-box have been observed in AtPR5 and AtPR12. These were observed to be highly expressed during seed germination. AtPR1, AtPR2 and AtPR5 were up-regulated after wounding; this may due to the presence of wound responsive element (W-box) in their promoter regions. AtPR1 and AtPR2 are highly expressed under drought stress condition which maybe because of AtMYC2 element, which acts as a drought inducible element.

TGACG-motif present in AtPR5, AtPR9, AtPR10, OsPR2, OsPR5, OsPR9 and OsPR12 increases the production of secondary metabolites by arresting or delaying cell cycle in G1/S checkpoint. This motif tends to be present in the promoter region of Kip-related proteins (cell cycle inhibitor) and inhibits the active Cyclin-dependent kinase/Cyclin complex [90]. The combined data of cis-element and the meta-analysis of PRs provided insights into the role of many PRs, and hence this information can be employed in future studies of PRs from other plant species.

## Conclusion

PR proteins play an important role in providing resistance to plants. Till now, no work has been reported on cis-elements present in *A*. *thaliana* and *O*. *sativa* PRs. Therefore, in the present work, an *in-silico* approach was followed to study the presence of cis-elements in PR genes. We also tried to validate our *in-silico* work with the wet lab studies wherever available. This work throws light on the promoter regions of PRs, which further can provide new ways for the plant genetic engineering technology for protection of crops against diseases.

## Supporting information

S1 Fig Pearson correlation analysis for gene lengths of AtPRs and OsPRs. (TIFF)

S1 Table Percentage similarity between AtPRs and OsPRs. (DOCX)

S2 Table CAREs along with their function conserved in both AtPRs and OsPRs; unique in AtPRs and unique in OsPRs. (DOCX)
