# Supplementary figures and images for: In-silico analysis of cis-acting regulatory elements of pathogenesis-related proteins of Arabidopsis thaliana and Oryza sativa
Source: PLoS One. 2017 Sep 14;12(9):e0184523. doi: 10.1371/journal.pone.0184523 (PMC5598985; doi:10.1371/journal.pone.0184523)

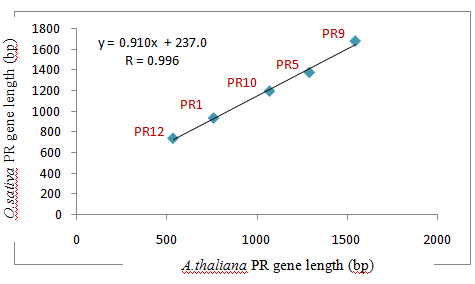

Supplement: S1 Fig — (TIFF) [file pone.0184523.s001.tiff]
