# Supplementary material for: In-silico analysis of cis-acting regulatory elements of pathogenesis-related proteins of Arabidopsis thaliana and Oryza sativa
Source: PLoS One. 2017 Sep 14;12(9):e0184523. doi: 10.1371/journal.pone.0184523 (PMC5598985; doi:10.1371/journal.pone.0184523)
Supplement: S1 Table — (DOCX) [file pone.0184523.s002.docx]

**Table S1.** Percentage similarity between AtPRs and OsPRs.

| AtPRs | OsPRs | % similarity |
| --- | --- | --- |
| AtPR1 | OsPR1 | 66.7 |
| AtPR2 | OsPR2 | 67.3 |
| AtPR5 | OsPR5 | 50 |
| AtPR9 | OsPR9 | 56.9 |
| AtPR10 | OsPR10 | 39.4 |
| AtPR12 | OsPR12 | 41.3 |
