# Supplementary material for: In-silico analysis of cis-acting regulatory elements of pathogenesis-related proteins of Arabidopsis thaliana and Oryza sativa
Source: PLoS One. 2017 Sep 14;12(9):e0184523. doi: 10.1371/journal.pone.0184523 (PMC5598985; doi:10.1371/journal.pone.0184523)
Supplement: S2 Table — (DOCX) [file pone.0184523.s003.docx]

**Table S2.** CAREs along with their function conserved in both AtPRs and OsPRs; unique in AtPRs and unique in OsPRs.

| **Name of CARE present in both *A.thaliana* and *O.sativa*** | **Function** | **PRs in which CARE is present** |
| --- | --- | --- |
| CAAT box | Promoter and enhancer region | PR1, PR2, PR5, PR9, PR10, PR12 |
| G-Box | Light responsiveness | PR1, PR5, PR9,PR10, PR12 |
| TATA box | Core promoter element | PR1, PR2, PR10, PR12 |
| MBS | MYB binding site involved in drought-inducibility | PR2, PR5, PR9, PR12 |
| Skn-1_motif | Required for endosperm expression | PR5, PR9, PR10, PR12 |
| Box 4 | Light responsiveness | PR1, PR2 |
| ABRE | Abscisic acid responsiveness | PR1, PR5 |
| Circadian | Involved in circadian control | PR2, PR10 |
| Sp1 | Light responsiveness | PR1, PR5 |
|  |  |  |
| GAG-Motif | Light responsiveness | PR2, PR10 |
|  |  |  |
| CGTCA-motif  GCC box  TGACG-motif | Involved in MeJA-responsiveness | PR5, PR9 |
| Unnamed_1, Unnamed_4 | Unknown function | PR1 |
| TC-rich repeats | Involved in defense and stress responsiveness | PR2 |
| ACE  O2-site | Light responsiveness  Involved in zein metabolism regulation | PR10 |
|  | | |
| **Name of CARE protein present only in *O.sativa*** | **Function** | **PRs in which CARE is present** |
| A box | Meristem-specific activation | PR1, PR5, PR9, PR10, PR12 |
| AC-I | Vascular-specific expression | PR2 |
| ATCT motif | Light responsiveness | PR10 |
| Box II | Light responsiveness | PR5 |
| CG motif | Anoxic specific inducibility | PR5, PR10 |
| CCAAT box | MYBHv1 binding site | PR2, PR5, PR9 |
| CCGTCC box | Meristem-specific activation | PR1, PR5, PR9, PR10, PR12 |
| CTAG motif | Critical de-terminant for repressor binding | PR12 |
| Chs-Unit 1 m1 | Light responsiveness | PR10 |
| CE 1 | Involved in ABA responsiveness | PR12 |
| EIRE | Elicitor responsive element | PR5 |
| GAG motif | Light responsiveness | PR1, PR2, PR10, PR12 |
| Gap box | Light responsiveness | PR10 |
| GARE motif | Gibberellins responsive element | PR9, PR12 |
| H box | Essential for chalcone synthase promoter | PR10 |
| Motif I | Root specific element | PR2 |
| Motif II b | Abscisic acid responsive | PR2, PR9 |
| Motif lib | Abscisic acid responsive | PR5 |
| MNF1 | Light responsiveness | PR9 |
| TATC box | Gibberellin responsive element | PR2 |
|  | | |
| **Name of CARE protein present only in *A.thaliana*** | **Function** | **PRs in which CARE is present** |
| AAGAA motif | Unknown function | PR9, PR12 |
| AC II | Vascular-specific expression | PR1, PR12 |
| ARE | Essential for anaerobic induction | PR9, PR10 |
| AT 1-motif | Light responsiveness | PR10 |
| ATC motif | Light responsiveness | PR5 |
| ATGCAA | Associated to TGAGTCA motif | PR1 |
| Aux RR Core | Involved in auxin responsiveness | PR9 |
| Box I | Light responsiveness | PR10 |
| Box W1 | Light responsiveness | PR9, PR10 |
| Chs-CMA1a | Light responsiveness | PR10 |
| EIRE | Elicitor-responsive element | PR10 |
| F box | Containing heat, drought, salicylic acid, and abscisic acid responsive cis-elements were able to respond to the abiotic stresses | PR9 |
| GA motif | light responsive element | PR10 |
| GCN4 motif | Involved in endosperm expression | PR12 |
| GATA-motif | Light responsiveness | PR10 |
| GT1 motif | Light responsiveness | PR10, PR12 |
| JERE | MeJA-responsiveness | PR10 |
| LTR | Low temperature responsiveness | PR10 |
| MBS | Involved in drought-inducibility | PR1, PR2, PR5, PR9, PR12 |
| MBSI | Flavonoid biosynthetic genes regulation | PR2 |
| TGA element | Auxin-responsive element | PR10 |
| Unnamed_3 | Unknown function | PR1, PR2, PR10 |
| W box | Elicitor-responsive element | PR9, PR10 |
